# Variables related to perceived stress and resilience among international migrants: a multicenter study (AFFAIR Project)

**DOI:** 10.1590/1980-220X-REEUSP-2024-0222en

**Published:** 2024-11-25

**Authors:** Mayckel da Silva Barreto, Maria do Céu Barbieri-Figueiredo, Francisca Maria Garcia-Padilla, Raquel Saenz Mendia, Renan Alves Silva, Florinda Laura Ferreira Rodrigues Galinha De Sá, Camila Aparecida Pinheiro Landim Almeida, Maria Joana Campos, Fernanda Lise, Sonia Silva Marcon

**Affiliations:** 1 Universidade Estadual de Maringá, Departamento de Enfermagem, Maringá, PR, Brazil.; 2 Universidad de Huelva, Facultad de Enfermería, Huelva, HU, Espanha.; 3 Universidad Pública de Navarra, Faculdad de Ciencias de la Salud, Departamento de Enfermería, Pamplona, NA, Espanha.; 4 Universidade Federal de Campina Grande, Departamento de Enfermagem, Campina Grande, PB, Brazil.; 5 Escola de Enfermagem de Lisboa, Lisboa, LI, Portugal.; 6 Universidade Católica Portuguesa, Faculdade de Enfermagem, Porto, PO, Portugal.; 7 Escola Superior de Enfermagem do Porto, PO, Portugal.; 8 Universidade Federal de Pelotas, Faculdade de Enfermagem, Departamento de Enfermagem, Pelotas, RS, Brazil.

**Keywords:** Resilience, Psychological, Stress, Psychological, Transients and Migrants, Emigration and Immigration, Migrant-Receiving Society, Multicenter Study, Resiliencia Psicológica, Estrés Psicológico, Migrantes, Emigración e Inmigración, Sociedad Receptora de Migrantes, Estudio Multicêntrico

## Abstract

**Objective:**

To identify variables related to perceived stress and resilience of international migrants.

**Method:**

Multicenter, observational, cross-sectional study carried out with 403 migrants residing in Brazil, Spain, or Portugal. The following instruments were used to collect data: *Perceived Stress Scale* and *Resilience Scale*. Student's t-test and analysis of variance were applied in the analysis.

**Results:**

Perceived stress was related to: living in Brazil or Portugal; shorter stay in the host country; being black or brown; and having no religion. Greater resilience was related to: not being divorced; having less education and lower family income; being from developing countries; living in Spain; and having a religion.

**Conclusion:**

Aspects such as host and origin country, skin color, income, marital status, education, length of stay in the host country, and religion are related to the level of stress and/or resilience of migrants. Knowing this profile is useful for developing public integration policies and interventions that seek to reduce stress and improve resilience.

## RESUMEN

**Objetivo:**

Identificar variables relacionadas con el estrés percibido y la resiliencia de los migrantes internacionales.

**Método:**

Estudio multicéntrico, observacional y transversal, realizado con 403 inmigrantes que vivían en Brasil, España o Portugal. Para la recolección de datos se utilizaron los siguientes instrumentos: *Escala* de *Estrés Percibido* y *Escala de resiliencia*. En el análisis se aplicó la prueba t de Student y análisis de varianza.

**Resultados:**

El estrés percibido se relacionó con: vivir en Brasil o Portugal; estancia más corta en el país anfitrión; ser negro o marrón; y no tener religión. La mayor resiliencia estuvo relacionada con: no estar divorciado; tener menos educación y menores ingresos familiares; ser de países en desarrollo; vivir en España; y tener religión.

**Conclusión:**

Aspectos como el país de acogida y el país de origen, el color de la piel, los ingresos, el estado civil, la educación, la duración de la estancia en el país de acogida y la religión están relacionados con el nivel de estrés y/o resiliencia de los migrantes. Conocer este perfil es útil para desarrollar políticas e intervenciones públicas de integración que busquen reducir el estrés y mejorar la resiliencia

## INTRODUCTION

Immigration and refuge are contemporary and global phenomena. They reach a significant number of people who choose to leave their country of origin in search for better living conditions (immigrants) or are forced to leave their homes due to the adverse effects of climate change, environmental disasters, racial and religious intolerance, xenophobia, and wars (refugees)^(1,2)^. This study adopted the broad term “international migrant”, also used by the International Organization for Migration (IOM), with a view to recognizing the migrant (whether immigrant or refugee) as an individual with rights. It is therefore a question of focusing mainly on migration as a complex, vulnerability trigger human-social phenomenon, which necessarily involves crossing different territories, whether national or transnational^(1)^.

In 2022, it was estimated that there were around 400 million migrants in the world, the largest contingent since World War II^(2,3)^. This dramatic situation, experienced amid the social, economic, and political repercussions of the Covid-19 pandemic, has contributed to increased insecurity, fear, worsening of physical and mental health, and increased stress levels among international migrants. Consequently, there has also been serious well-being impairment of these people and violation of their basic rights^(4)^.

It is known that the migration process is a key determinant for health, with international migrants being more exposed to vulnerabilities and suffering physical and/or psychological trauma^(3)^. This is because there is constant exposure to various stressful events, such as fleeing dangerous routes, sleep deprivation, malnutrition, forced separation from family, permanent feelings of insecurity and fear, stigmatization, and prejudice. All of these aspects can be recognized by the body as a threat to homeostasis or balance, increasing stress levels and negatively impacting physical and mental health^(4,5)^.

Studies conducted with different groups of international migrants, including Latin women^(6)^, young adults^(7)^, middle-aged^(8)^ or older^(9)^ adults, and those who lived in culturally diverse countries such as China^(7.9)^, Israel^(10)^ and United States^(6)^ showed that the highest level of perceived stress affected different aspects of daily life, hindering social integration and the maintenance of well-being and sleep quality^(6-9)^. Stress was associated with the profile of younger, single migrants who lived alone and who perceived themselves as less resilient^(9,10)^.

Despite exposure to great adversity/vulnerabilities, evidence has suggested that international migrants are capable of becoming resilient^(11,12)^. To deal with significant sources of stress, it is important that people in the migration process and their families develop resilience – the ability to adapt to adversity. It occurs especially through mental, emotional, and behavioral flexibility and adjustment to external and internal demands. Resilience, therefore, is configured as a positive characteristic of the individual personality, which increases the possibility of adapting to or overcoming adversity, but which is also mediated by culture and society^(13,14)^.

Research has shown that developing resilience provides a better quality of life^(11)^ and greater self-efficacy and self-esteem^(12)^. Furthermore, it has worked as a protective factor for mental health, by reducing levels of post-traumatic stress disorder, anxiety and depression^(11)^. and by allowing the establishment of strong connections to social networks of family and friends and the strengthening of faith and religion^(13,15,16)^. The aspects that seem to favor resilience and, at the same time, reduce stress, involve the maintenance of cultural identity, the provision of social support and the development of a sense of belonging and security in the host country^(9,10,12)^.

The results of these studies on stress and resilience showed that different variables are related to these outcomes. Variables are understood as the differences that a given population under study presents, such as sex or gender, age, weight, height, marital status, and socioeconomic status^(17)^. It should be noted that current research has not yet addressed the influence of the Latin American migration process on stress and resilience, both in relation to the South-South axis (regional migration) or to Latin migration, for the European context. Furthermore, studies are still scarce given the large number of international migrants that exist today and the multiplicity of causal contexts of migration and possible reception environments. In this regard, we recognize the need to continue investigating, in multiple scenarios, how the migration process has influenced people’s stress and resilience. This is relevant for planning social and health service systems, as well as for preparing professionals who work with international migrants. Professional, culturally sensitive, and competent action can propose safe interventions that mitigate stress, promote resilience and allow the migrant to integrate into the host society^(18)^.

Therefore, the question is: what are the variables related to perceived stress and resilience of international migrants? Given the above, the present study aimed to identify variables related to perceived stress and resilience of international migrants.

## METHOD

### Design of Study

Multicenter, observational, and cross-sectional study, which used the STROBE (*Strengthening the Reporting of Observational studies in Epidemiology*) tool as a guide for its description.

### Population and Local

Currently, Portugal, Spain, and Brazil represent important host countries for migrants. In Portugal, in 2021, there were 698,887 documented international citizens, that is, 7% of the population, the highest value recorded by the Foreigners and Borders Service since its creation in 1976^(2)^. In Spain, the number of migrants with residence documentation in June 2022 was 6,246,130, which corresponded to 13% of the population. In the last half of 2022, there was a 128% increase in the number of Ukrainian migrants and a 9% increase in the number of Colombians^(3)^. Brazil, in turn, has been receiving constant influx of people from countries such as Venezuela, Colombia, and Haiti. In 2021, around 1.4 million migrants resided in the country and, in ten years, there was a 24.4% increase in the number of new registered migrants^(19)^.

The investigation was carried out with international migrants, regardless of their country of origin, from a non-probabilistic sample of people living in the following three cities in each of the countries mentioned: Maringá, Pelotas and Boa Vista (Brazil); Pamplona, Huelva, and Toledo (Spain); Funchal, Lisbon, and Porto (Portugal). The inclusion criteria adopted were: being an international migrant over 18 years of age and having resided for a minimum of 6 months and a maximum of 10 years in the host country. On the other hand, 12 people who were not proficient in reading, understanding, and communicating in Portuguese or Spanish were excluded.

### Variables Under Study and Instruments Used

Data were collected through the application of the following instruments: a) questionnaire regarding sociodemographic variables; b) Perceived Stress Scale-14 (PSS-14); and c) Personal Resilience Scale.

a) Sociodemographic questionnaire: prepared by the researchers, consisting of the following variables: gender (male, female, or other); age (in complete years of life); marital status (with partner and without partner); nationality; length of stay in the host country (counted in months or years); level of education (complete years of study); average family income (sum of the income of cohabiting family members, divided by the number of people. Variable measured in euros); religion (religious practice with which the person identifies); occupation (paid activity(ies) performed); and skin color (self-declared by the participant: white, black, brown, yellow or indigenous).

b) *Perceived Stress Scale* (PSS-14): measures the intensity with which people perceive situations as stressful^(20)^, which is translated and validated for the Brazilian, Portuguese, and Spanish contexts [source omitted due to limitation in the number of references allowed]. Respondents indicated their responses using a Likert-type scale, with five possible answers ranging from “never” to “very often”. The total result varies between 0 and 56 points, with items 4, 5, 6, 7, 9, 10, and 13 having inverse scores^(20)^.

c) *Resilience Scale* (RS): validated for the Brazilian, Portuguese and Spanish contexts [source omitted due to limitation on the number of references allowed], with a seven-point Likert scale (“completely disagree” to “completely agree”). Total scores range from 25 to 175, with higher scores indicating a high degree of resilience^(21)^.

It is worth noting that the scale scores do not have a cut-off point or classification. The average was used to divide the sample between: “more stressed and less stressed” and “more resilient and less resilient”.

### Period and Data Collection

Contact with potential participants began through religious groups and Non-Governmental Organizations (NGOs) that receive international migrants, located in three cities in different regions of each of the countries under study. These entities, at their headquarters, provide various services to this population and the researchers inserted themselves into these contexts to approach and invite migrants to participate in the study.

Data collection, conducted by a group of previously trained researchers, took place between January and September 2022, through structured interviews, always conducted in Portuguese or Spanish and carried out in person at the NGOs’ headquarters or at the migrants’ homes. At the end of each interview, the participant was invited to indicate one or more migrants they knew, providing their address and/or telephone number. New potential participants were contacted, preferably by telephone, to present the objectives of the study, verify the inclusion criteria and invite them to participate. If they accepted, a home visit was scheduled on a suitable day and time.

Thus, the selection of participants was characterized as non-probabilistic, due to the researchers’ convenience of access, of the snowball type. Given the limited time and human and financial resources to continue with data collection, the sample reached 403 individuals.

### Data Processing and Statistical Analysis

All information was entered into an Excel for Windows 2010 spreadsheet^®^ and after double checking, exported to the statistical software SPSS, version 22, for processing the analyses. Cronbach’s Alpha test showed that the Resilience Scale (0.933) was considered “excellent” and the Perceived Stress Scale (0.712) “acceptable” for the sample investigated.

From the Kolmogorov-Smirnov and Shapiro-Wilk normality tests, it was found that the data followed a normal distribution. To identify the differences between the means, Student’s t-test and analysis of variance (ANOVA) tests were used, with Sidak’s post-hoc test being performed to evaluate the multiple comparisons that were statistically significant in the mean difference tests. The confidence interval was 95% and the significance level adopted was 5% for all tests (p ≤ 0.05).

### Ethical Aspects

The research was developed in accordance with research ethics guidelines and its project was submitted for approval by evaluation committees in the three countries (Brazil: 4,450,114; Spain: 2756-N21; Portugal: 001/2021). The researchers ensured free participation, confidentiality of information, and anonymity of the interviewees who signed the Free and Informed Consent Form in two copies.

## RESULTS

A total of 403 international migrants participated in the study. The majority were female (65.0%); aged between 35 and 59 years (50.2%); had more than eight years of education (78.5%); had a partner (54.4%); had a monthly family income *per capita* of more than 100 euros (61.3%); was from South America (58.6%); lived in Brazil (40.4%); had a paid job (60.8%); declared him/herself black/brown (41.2%); lived with up to four people (65.8%); and was Catholic/Christian (64.8%). It should be noted that 84.9% had immigrated, at maximum, five years before (Table 1), with an average of 28 months in Portugal, 30.1 months in Brazil, and 35.3 months in Spain.

**Table 1:** Distribution of variables according to averages of perceived stress and resilience among international migrants in Brazil, Spain, and Portugal, 2022.

VARIABLES	N	%	Perceived stress	p-value	Resilience	p-value
**Sex**						
Female	262	65.0	24.64	0.688	137.87	0.089
Male	141	35.0	24.95		133.73	
**Age**						
18–34 years old	192	47.6	24.77	0.590	135.49	0.352
35–59 years old	202	50.2	24.75		137.54	
60 or more years old	09	2.2	24.22		134.77	
**Level of Education**						
Up to 8 years of study	87	21.6	23.88	0.102	142.35	<0.001*
More than 8 years of study	316	78.5	24.98		134.81	
**Marital status**						
Single	160	39.7	24.88	0.733	139.48	0.004*
Married/Common law marriage	219	54.4	24.63		137.68	
Divorced	19	4.7	24.99		125.89	
Widower	05	1.2	24.43		138.80	
**Family income per capita**						
Up to 100 euros^†^	156	38.7	24.60	0.889	141.14	0.003*
More than 100 euros	247	61.3	24.83		133.10	
**Nationality**						
South America	236	58.6	24.54	0.155	137.04	<0.001*
Africa	80	19.8	24.83		136.71	
Asia	03	0.7	34.00		99.33	
Europe	35	8.7	25.37		127.22	
Central America	47	11.7	26.35		144.23	
North America	02	0.5	24.28		88.00	
**Receiving country**						
Brazil	163	40.4	25.54	<0.001*	137.22	<0.001*
Spain	140	34.7	22.72		144.80	
Portugal	100	24.8	26.28		123.46	
**Migration time**						
Up to 5 years	342	84.9	25.96	<0.001*	136.92	0.090
More than 5 years	61	15.1	21.07		133.70	
**Occupation**						
Unemployed	91	22.6	24.89	0.903	135.58	0.713
Employee/self-employed	245	60.8	24.79		137.18	
Home/student	67	16.6	24.38		134.89	
**Skin color**						
Black/Brown	166	41.2	26.18	0.006*	148.46	<0.001*
White	163	40.4	24.12		135.39	
Indigenous	61	15.1	22.98		147.65	
Yellow	13	3.2	22.46		132.40	
**Number of cohabitants**						
Up to 4 people	265	65.8	24.72	0.815	135.90	0.125
5 or more people	138	34.2	24.80		137.47	
**Religion**						
Catholicism/Christianity	261	64.8	24.31	0.001*	139.67	<0.001*
None	86	21.3	26.91		122.08	
Orthodox	39	9.7	21.91		145.66	
Islam	12	3.0	23.25		146.15	
Other	05	1.1	23.60		137.00	

*Significant p-value in Student’s T-Test (two categories) or analysis of variance (three categories or more);

^†^euro exchange rate on 12/27/2023: R$5.37.

It was found that perceived stress was significantly higher among migrants living in Brazil or Portugal (p < 0.001); who had immigrated less than five years before (p < 0.001); had brown/black skin color (p: 0.006); and reported having no religion (p < 0.001).

Resilience was greater among migrants with up to eight years of education (p < 0.001); single, married or widowed (p: 0.004); who had a monthly family income *per capita* of up to 100 euros (p: 0.003); who lived in Spain (p < 0.001); and originating from South America, Central America, and Africa (p < 0.001). Furthermore, migrants of European origin had a higher average resilience compared to those from North America and Asia (p < 0.001).

Migrants of black/brown race/color and indigenous origin had greater resilience compared to migrants of yellow (Asian origin) and white (p < 0.001) skin color. Finally, it was found that those who declared having some religion presented higher resilience averages (p < 0.001).

## DISCUSSION

The study showed that different variables were related to higher levels of perceived stress and resilience among international migrants. The fact that it was demonstrated that individuals living in Spain were more resilient and those living in Brazil and Portugal had higher levels of stress is noteworthy, since data from MIPEX Group - *Migrant Integration Policy Index* indicate that of these three countries, Spain has the worst migrant integration rates, with the exception of access to health services^(22)^. However, the same data indicate that, in Brazil, aspects such as access to education, health, and political participation are well below the global average^(22)^, which is known to increase stress^(9-11)^. In Portugal, there is also significant hate speech/xenophobia involving migrants. Data from the MigraMyths project showed that more than 75% of the migrants interviewed who lived in Portugal had already suffered prejudices and stereotypes linked to migration^(23)^. Furthermore, the growth of the radical right-wing populism in Brazil and Portugal (unlike Spain to date) has also been highlighted as a relevant factor in the dissemination of hate speech against racial and ethnic minorities, which even calls for the expulsion of groups considered foreign^(23)^. In view of this, it is assumed that the absence of a political context that exposes migrants to stressful situations, associated with a longer average length of stay in the host country, may be contributing to better integration and also to the development of resilience among those living in Spain.

In this regard, the need for rapid and intense adaptation to the new environment may justify the fact that perceived stress was greater among those who had immigrated less than five years ago. A person who migrates to a new country faces a variety of changes and obstacles, including adapting to the new culture, language barriers, finding a job, acquiring housing, and forming new social networks^(24)^. People may also experience feelings of isolation, loneliness, and uncertainty as a result of being in an unfamiliar environment and often away from their family and social support networks^(25)^. In addition, unfamiliarity with the legal system, bureaucracy, difficulty entering the job market, and restricted access to basic services can cause stress^(26)^.

Data from the present study demonstrated that brown and black people perceived themselves as more stressed and at the same time more resilient. Furthermore, migrants from South America, Central America, and Africa also showed higher levels of resilience. These data may overlap given that migrants from these regions are mostly black^(4,19)^. Greater exposure to xenophobia associated with racial prejudice can act as a trigger for stress and, to the same extent, lead the individual to use strategies to overcome difficulties, thus perceiving themselves as more resilient to the numerous challenges they face during the process of integration into the new society^(4,5)^.

It is known that resilience is influenced by culture^(14)^. Thus, the perception of social support according to the migrants’ place of origin may be due to cultural experience and the type of family network formed. In the study in question, Latin Americans were the most resilient, possibly because they belong to collectivist-horizontal cultures, in which sociability among equals is promoted compared to other more individualistic cultures, which tend to present lower values of family and social support^(27)^.

It is also worth noting that some Latin and African cultures have customs and values that consider family, community and solidarity as strategies for overcoming challenges^(4,5)^. Migrants can use these principles as a social and emotional foundation to endure adversity. Due to economic, political, conflict or other circumstances, a large number of Latin and African migrants leave their countries of origin in search of better perspectives^(1,2)^. Faced with the need to survive and ensure a better future for themselves and their families, they tend to develop resilience^(5)^.

However, it is essential to avoid overgeneralizations and not consider resilience as a universal characteristic for all Latin and African migrants. The experience of international migrants is different for everyone and resilience is not uniform across all, regardless of their ethnic or cultural background. Furthermore, a variety of individual and contextual characteristics can influence resilience, as it is not genetic but rather a context-related skill^(13,14)^.

This study showed that divorced people were less resilient. The fact that couples go through a divorce, which breaks the family structure, can be a factor in making them less resilient, even more so if this event occurs in the country to which they migrated. In addition to all the challenges of migration and asylum in a new country, having to experience another family transition, which creates a new rupture with established interactions, previous roles, and the family and social support network, can hinder resilience^(28)^ development.

The connection between better adaptation results for migrants and the presence of adequate family functionality has already been established in the literature. For instance, migrant families’ involvement in their children’s education has been shown to be beneficial to their children’s academic success^(29)^. This mutual aid can be especially useful for people facing family problems such as conflicts and divorce, as well as discrimination or other obstacles to integration. However, it is important to note that not all migrant families have the same resources or opportunities to contribute positively to their outcomes^(30)^. The experiences of migrant families can be shaped by variables such as social and economic position, language skills and access to social support networks. It is essential that health professionals and managers take these differences into account when creating policies, programs, or interventions that seek to promote care for migrant communities.

Finally, the results of the present study suggest that the absence of religiosity is related to higher levels of stress and lower levels of resilience. Despite the divergent views and opinions of many scientists and mental health professionals, research examining religion, spirituality, and health has expanded rapidly^(31)^.

Different studies, with mixed methods, which investigated the resilience in Mexican migrants^(14)^, Latinos(16) and Africans^(5)^ living in the USA, identified that the most resilient migrants were those who had high levels of religiosity and used faith as a coping strategy to make sense of their difficulties and suffering. Similarly, quantitative investigations conducted with 128 Latin migrants in the USA^(15)^ and with 154 young Moroccan migrants in Spain^(20)^ showed that resilience was directly related to religiosity. In the meantime, it is clear that promoting social support and religion in migrant communities can improve well-being by increasing resilience and reducing stress and suffering.

This multicenter study advances knowledge by demonstrating that international migrants, in different contexts, experience perceived stress but, in return, are able to become resilient. These findings can help managers and health and social assistance professionals to think about public policies that seek to integrate international migrants into society, considering the profile of the migrant who perceives stress the most and who, therefore, needs to develop resilience more fiercely. Therefore, nurses can benefit from developing targeted intervention strategies, especially for migrants who have been in the host country for a shorter period of time, who have black or brown skin color, and who have no religion, to promote resilience by strengthening mental health, developing the ability to adapt to stressors and changes and learning/using self-control, positivity, and optimism.

In contrast, it is recognized that the study has limitations. One of them refers to the use of non-probabilistic sampling, from three cities in each participating country. Another is related to the fact that more than 70% of those interviewed are from Latin America. Such characteristics circumscribe the results found and, therefore, caution is suggested when comparing them with other migration scenarios and contexts.

Nonetheless, the findings are important in enabling healthcare professionals to support and enhance organizations’ efforts to advocate and encourage the formulation of legislation and policies aimed at reducing stress and increasing resilience among migrants. Policymakers should focus on additional resources to improve the physical and mental well-being of international migrants. This has the potential to impact health, improving their insertion and integration into society and reducing the use of health services, which contributes to the sustainability of the health systems in host countries^(14)^. Based on the diagnosis raised, future studies should focus on developing interventions directed to reducing stress and promoting resilience among people and families experiencing the migration process.

## CONCLUSION

The results of this study allowed us to observe that the variables related to the highest level of perceived stress were: living in Brazil or Portugal; time spent in the host country below five years; being black or brown; and having no religion. The variables associated with greater resilience were: not being divorced; having up to eight years of education; having a montlhy family income *per capita* of up to 100 euros; being from Central America, South America, and Africa; living in Spain; and having a religion.

## References

[1] Graf S, Rubin M, Assilamehou-Kunz Y, Bianchi M, Carnaghi A, Fasoli F (2023). Migrants, asylum seekers, and refugees: different labels for immigrants influence attitudes through perceived benefits in nine countries. Eur J Soc Psychol.

[2] UNHCR (2022). Global Trends Forced Displacement in 2022.

[3] World Health Organization (2022). World report on the health of refugees and migrants: summary.

[4] Lee J, Hong J, Zhou Y, Robles G (2020). The relationships between loneliness, social support, and resilience among Latinx immigrants in the United States. Clin Soc Work J.

[5] Corley A, Sabri B (2021). Exploring African immigrant women’s pre- and post-migration exposures to stress and violence, sources of resilience, and psychosocial outcomes. Issues Ment Health Nurs.

[6] Ryan D, Tornberg-Belanger SN, Perez G, Maurer S, Price C, Rao D (2021). Stress, social support and their relationship to depression and anxiety among Latina immigrant women. J Psychosom Res.

[7] Xia Y, Ma Z (2020). Social integration, perceived stress, locus of control, and psychological wellbeing among chinese emerging adult migrants: A conditional process analysis. J Affect Disord.

[8] Chen R, Slopen N, Lee S (2023). Perceived stress, recent stressors, and distress in relation to sleep disturbance and duration among middle-aged and older Asian immigrants. Sleep Health.

[9] Wang H, Hou Y, Zhang L, Yang M, Deng R, Yao J (2022). Chinese elderly migrants’ loneliness, anxiety and depressive symptoms: the mediation efect of perceived stress and resilience. Front Public Health.

[10] Zlotnick C, Dryjanska L, Suckerman S (2022). Health literacy, resilience and perceived stress of migrants in Israel during the COVID-19 pandemic. Psychol Health.

[11] Kong LN, Zhang N, Yuan C, Yu ZY, Yuan W, Zhang GL (2021). Relationship of social support and health-related quality of life among migrant older adults: the mediating role of psychological resilience. Geriatr Nurs.

[12] Mattelin E, Paidar K, Söderlind N, Fröberg F, Korhonen L (2024). A systematic review of studies on resilience and risk and protective factors for health among refugee children in Nordic countries. Eur Child Adolesc Psychiatry.

[13] Torres SM, Lusk M (2018). Factors promoting resilience among Mexican immigrant women in the United States: applying a positive deviance approach. Estud Front (Méx).

[14] Wiig S, Aase K, Billett S, Canfield C, Roise O, Nja O (2020). Defining the boundaries and operational concepts of resilience in the resilience in healthcare research program. BMC Health Serv Res.

[15] Revens KE, Gutierrez D, Paul R, Reynolds AD, Price R, Dehaven MJ (2021). Social support and religiosity as contributing factors to resilience and mental wellbeing in latino immigrants: a community-based participatory research study. J Immigr Minor Health.

[16] Lusk M, Terrazas S, Caro J, Chaparro P, Antúnez DP (2019). Resilience, faith, and social supports among migrants and refugees from Central America and Mexico. J Spiritual Ment Health.

[17] Bonita R, Beaglehole R, Kjellström T (2010). Epidemiologia básica.

[18] World Health Organization (2023). Promoting the health of refugees and migrants.

[19] Cavalcanti L, Oliveira T, Silva BG (2022). Relatório Anual OBMigra 2022.

[20] Lee EH (2012). Review of the psychometric evidence of the perceived stress scale. Asian Nurs Res.

[21] Wagnild GM, Young HM (1993). Development and psychometric evaluation of the Resilience Scale. J Nurs Meas.

[22] Grupo MIPEX (2023). Migrant Integration Policy Index.

[23] Costa AP (2021). Discurso de ódio e imigração em Portugal.

[24] Lefrid M, Torres EN, Okumus F (2022). Immigrant hospitality workers: Familism, acculturation experiences, and perception of workplace. Int J Hospit Manag.

[25] Moraes MCL, Araújo LCN, Camargo CL (2023). Black immigrants in São Paulo-Brazil: sociodemographic profile, reason for coming, embracement, and health. Rev Esc Enferm USP.

[26] Pizzol ESRD, Barreto MS, Barbieri-Figueiredo MC, Ugarte-Gurrutxaga MI, Garcia-Padilla FM, Santos ML (2023). Perspective of immigrants on personal and family integration in brazilian society. Texto Contexto Enferm.

[27] Henríquez D, Urzúa A, López-López W (2022). Social support as a mediator of the relationship between identity fusion and psychological well-being in south-south migrant populations. J Int Migr Integr.

[28] Cox RB, Brosi M, Spencer T, Masri K (2021). Hope, stress, and post-divorce child adjustment: development and evaluation of the co-parenting for resilience program. J Divorce & Remarriage.

[29] Morassaei S, Irvin E, Smith PM, Wilson K, Ghahari S (2022). The role of immigrant admission classes on the health and well-being of immigrants and refugees in Canada: A scoping review. J Immigr Minor Health.

[30] Hamari L, Konttila J, Merikukka M, Tuomikoski AM, Kuoven P, Kurki M (2022). Parent support programmes for families who are immigrants: A scoping review. J Immigr Minor Health.

[31] Schwalm FD, Zandavalli RB, Castro ED, Lucchetti G (2022). Is there a relationship between spirituality/religiosity and resilience? A systematic review and meta-analysis of observational studies. J Health Psychol.

