# Supplementary material for: Variables related to perceived stress and resilience among international migrants: a multicenter study (AFFAIR Project)
Source: Rev Esc Enferm USP. 2024 Nov 25;58:e20240222. doi: 10.1590/1980-220X-REEUSP-2024-0222en (PMC11623441; doi:10.1590/1980-220X-REEUSP-2024-0222en)
Supplement: Translation

# Translation: Variáveis relacionadas ao estresse percebido e à resiliência entre migrantes internacionais: estudo multicêntrico (AFFAIR Project)

**DOI:** 10.1590/1980-220X-REEUSP-2024-0222pt

**Authors:** Mayckel da Silva Barreto, Maria do Céu Barbieri-Figueiredo, Francisca Maria Garcia-Padilla, Raquel Saenz Mendia, Renan Alves Silva, Florinda Laura Ferreira Rodrigues Galinha De Sá, Camila Aparecida Pinheiro Landim Almeida, Maria Joana Campos, Fernanda Lise, Sonia Silva Marcon

**Affiliations:** 1 Universidade Estadual de Maringá, Departamento de Enfermagem, Maringá, PR, Brasil.; 2 Universidad de Huelva, Facultad de Enfermería, Huelva, HU, Espanha.; 3 Universidad Pública de Navarra, Faculdad de Ciencias de la Salud, Departamento de Enfermería, Pamplona, NA, Espanha.; 4 Universidade Federal de Campina Grande, Departamento de Enfermagem, Campina Grande, PB, Brasil.; 5 Escola de Enfermagem de Lisboa, Lisboa, LI, Portugal.; 6 Universidade Católica Portuguesa, Faculdade de Enfermagem, Porto, PO, Portugal.; 7 Escola Superior de Enfermagem do Porto, PO, Portugal.; 8 Universidade Federal de Pelotas, Faculdade de Enfermagem, Departamento de Enfermagem, Pelotas, RS, Brasil.

**Keywords:** Resiliência Psicológica, Estresse Psicológico, Migrantes, Emigração e Imigração, Sociedade Receptora de Migrantes, Estudo Multicêntrico

## Abstract

**Objetivo:**

Identificar as variáveis relacionadas ao estresse percebido e à resiliência de migrantes internacionais.

**Método:**

Estudo multicêntrico, observacional e de corte transversal, realizado com 403 migrantes que residiam no Brasil, Espanha ou Portugal. Para a coleta de dados foram utilizados os instrumentos: *Perceived Stress Scale* e *Resilience Scale*. Na análise foram aplicados os testes t-student e análise de variância.

**Resultados:**

O estresse percebido esteve relacionado com: viver no Brasil ou em Portugal; menor tempo de permanência no país anfitrião; ser negro ou pardo; e não ter religião. Maior resiliência relacionou-se com: não ser divorciado; ter menor escolaridade e menor renda familiar; ser originário de países em desenvolvimento; viver na Espanha; e ter religião.

**Conclusão:**

Aspectos como país de acolhimento e de origem, cor da pele, renda, estado civil, escolaridade, tempo de permanência no país anfitrião e religião estão relacionados com o nível de estresse e/ou resiliência de migrantes. Conhecer esse perfil é útil para a elaboração de políticas públicas de integração e intervenções que busquem diminuir o estresse e melhorar a resiliência.

## INTRODUÇÃO

A imigração e o refúgio são fenômenos contemporâneos e globais. Atingem expressivo número de pessoas que optam por sair de seu país de origem em busca de melhores condições de vida (imigrantes) ou veem-se obrigadas a deixar suas casas em decorrência dos efeitos adversos das mudanças climáticas, desastres ambientais, intolerâncias raciais, religiosas, xenofobia e guerras (refugiados)^(1,2)^. O presente estudo adotou o termo abrangente “migrante internacional”, também utilizado pela Organização Internacional para Migração (OIM), tendo em vista o reconhecimento do migrante (seja ele imigrante ou refugiado) enquanto sujeito de direitos. Trata-se, então, de enfocar principalmente a migração como fenômeno humano-social, complexo e desencadeador de vulnerabilidades, que necessariamente envolve o atravessar diferentes territórios sejam eles nacionais ou transnacionais^(1)^.

Em 2022 estimava-se que no mundo havia cerca de 400 milhões de migrantes, o maior contingente desde a segunda guerra mundial^(2,3)^. Essa dramática situação, vivenciada em meio aos reflexos sociais, econômicos e políticos da pandemia de Covid-19, tem contribuído para o aumento da insegurança, medo, piora na saúde física e mental e elevação do nível de estresse entre migrantes internacionais. Por conseguinte, também tem havido grave comprometimento do bem estar dessas pessoas e violação de seus direitos básicos^(4)^.

Sabidamente o processo migratório é determinante-chave para a saúde, sendo que as pessoas migrantes internacionais estão mais expostas a vulnerabilidades e a sofrer traumas físicos e/ou psicológicos^(3)^. Isso porque há constante exposição a diversos eventos estressores, tais como fuga por rotas perigosas, privação do sono, má nutrição, separação forçada da família, permanente sensação de insegurança e medo, estigmatização e preconceito. Todos esses aspectos podem ser reconhecidos pelo organismo como uma ameaça à homeostase ou equilíbrio, aumentando o nível de estresse e impactando negativamente na saúde física e mental^(4,5)^.

Estudos realizados com diferentes grupos de migrantes internacionais, incluindo mulheres latinas^(6)^, adultos jovens^(7)^, de meia-idade^(8)^ ou idosos^(9)^ e que viviam em países culturalmente variados como China^(7,9)^, Israel^(10)^ e Estados Unidos^(6)^ mostraram que o maior nível de estresse percebido afetava diferentes aspectos da vida diária, dificultava a integração social, assim como a manutenção do bem-estar e da qualidade do sono^(6-9)^. O estresse esteve associado ao perfil de migrantes mais jovens, solteiros, que viviam sozinhos e que se percebiam menos resilientes^(9,10)^.

Apesar da exposição a grandes adversidades/vulnerabilidades, as evidências têm sugerido que migrantes internacionais são capazes de se tornar resilientes^(11,12)^. Para o enfrentamento das significativas fontes de estresse é importante que as pessoas em processo migratório e suas famílias desenvolvam a resiliência – capacidade de se adaptar às adversidades. Ela ocorre especialmente por meio da flexibilidade mental, emocional e comportamental e do ajuste às demandas externas e internas. A resiliência, portanto, configura-se como uma característica positiva da personalidade individual, que aumenta a possibilidade de adaptação ou superação das adversidades, mas que também é mediada pela cultura e pela sociedade^(13,14)^.

As pesquisas tem mostrado que desenvolver a resiliência proporciona melhor qualidade de vida^(11)^ e maior autoeficácia e autoestima^(12)^. Além disso, ela tem funcionado como fator de proteção para a saúde mental, ao reduzir níveis de transtorno do estresse pós-traumático, ansiedade e depressão^(11)^ e ao permitir o estabelecimento de fortes ligações a redes sociais de familiares e amigos e o fortalecimento da fé e da religião^(13,15,16)^. Os aspectos que parecem favorecer a resiliência e, ao mesmo tempo, reduzir o estresse, envolvem a manutenção da identidade cultural, a oferta de apoio social e o desenvolvimento da sensação de pertencimento e segurança no país de acolhimento^(9,10,12)^.

Os resultados desses estudos sobre o estresse e a resiliência mostraram que diferentes variáveis estão relacionadas a esses desfechos. Por variáveis entende-se as diferenças que uma determinada população em estudo apresenta, tais como sexo ou gênero, idade, peso, estatura, estado civil e condição socioeconômica^(17)^. Destaca-se que as pesquisas correntes ainda não abordaram a influência do processo migratório latino-americano, tanto relativo ao eixo sul-sul (migração regional), nem tampouco à migração latina, para o contexto europeu, sobre o estresse e a resiliência. Além disso, os estudos ainda são parcos tendo em vista o grande número de migrantes internacionais existentes hoje e a multiplicidade de contextos causais da migração e de ambientes de acolhimento possíveis. Nesse sentido, se reconhece a necessidade de seguir investigando, em múltiplos cenários, como o processo migratório tem influenciado o estresse e resiliência das pessoas. Isso é relevante para o planejamento dos sistemas de serviço social e de saúde, bem como para o preparo dos profissionais que atuam junto aos migrantes internacionais. A atuação profissional, culturalmente sensível e competente, pode propor intervenções seguras que mitiguem o estresse, promovam a resiliência e permitam a integração do migrante à sociedade acolhedora^(18)^.

Assim, questiona-se quais as variáveis relacionadas ao estresse percebido e à resiliência de pessoas migrantes internacionais? Diante do exposto, o presente estudo teve o objetivo de identificar as variáveis relacionadas ao estresse percebido e à resiliência de migrantes internacionais.

## MÉTODO

### Tipo do Estudo

Estudo multicêntrico, observacional e transversal, que utilizou como guia para sua descrição a ferramenta STROBE (*Strengthening the Reporting of Observational studies in Epidemiology*).

### Local e População

Na atualidade, Portugal, Espanha e Brasil representam importantes países de acolhida para migrantes. Em Portugal, no ano de 2021, havia 698.887 cidadãos internacionais documentados, ou seja, 7% da população, valor mais elevado registrado pelo Serviço de Estrangeiros e Fronteiras desde a sua criação em 1976^(2)^. Na Espanha, o número de migrantes com documentação de residência em junho de 2022 era de 6.246.130, o que correspondia a 13% da população. No último semestre de 2022, houve um crescimento de 128% no número de migrantes ucranianos e de 9% no número de colombianos^(3)^. O Brasil, por sua vez, tem recebido com frequência constante ingresso de pessoas originárias de países como Venezuela, Colômbia e Haiti. Em 2021, cerca de 1,4 milhão de migrantes residiam no país e, em dez anos, houve um aumento de 24,4% no número de novos migrantes registrados^(19)^.

A investigação ocorreu com migrantes internacionais, independente do país de origem, a partir de uma amostra não probabilística de pessoas que viviam em três cidades de cada um dos países, a saber: Maringá, Pelotas e Boa Vista (Brasil); Pamplona, Huelva e Toledo (Espanha); Funchal, Lisboa e Porto (Portugal). Os critérios de inclusão adotados foram: ser migrante internacional maior de 18 anos e residir no mínimo há 6 meses e no máximo 10 anos no país de acolhimento. Por outro lado, foram excluídas 12 pessoas que não apresentavam proficiência para leitura, compreensão e comunicação em língua portuguesa ou espanhola.

### Variáveis em Estudo e Instrumentos Utilizados

Os dados foram coletados a partir da aplicação dos seguintes instrumentos: a) questionário referente às variáveis sociodemográficas; b) Escala de Estresse Percebido-14 (PSS-14) e; c) Escala de Resiliência pessoal.

a) Questionário sociodemográfico: elaborado pelos pesquisadores, constituído pelas seguintes variáveis: gênero (masculino, feminino ou outro); idade (em anos completos de vida); estado civil (com companheiro(a) e sem companheiro(a)); nacionalidade; tempo de permanência no país anfitrião (contado em meses ou anos); escolaridade (anos completos de estudo); renda familiar média (soma da renda dos familiares que coabitam, dividida pelo número de pessoas. Variável medida em euros); religião (prática religiosa com a qual a pessoa se identifica); ocupação (atividade(s) remunerada(s) que exerce); e cor da pele (autodeclarada pelo participante: branca, negra, parda, amarela ou indígena).

b) *Perceived Stress Scale* (PSS-14): mede a intensidade com que as pessoas percebem as situações como estressantes^(20)^, a qual está traduzida e validada para os contextos brasileiro, português e espanhol [fonte omitida pela limitação no número de referências permitido]. Os entrevistados assinalaram as respostas por meio de uma escala do tipo Likert, com cinco possíveis respostas que variavam de “nunca” a “muito frequentemente”. O resultado total varia entre 0 e 56 pontos, sendo que os itens 4, 5, 6, 7, 9, 10 e 13 têm pontuação inversa^(20)^.

c) *Resilience Scale* (RS): validada para o contexto brasileiro, português e espanhol [fonte omitida pela limitação no número de referências permitido], com escala Likert de sete pontos (“discordo totalmente” a “concordo totalmente”). Os escores totais variam de 25 a 175, com os mais elevados indicando alto grau de resiliência^(21)^.

Destaca-se que o score das escalas não possui ponto de corte ou classificação. A média foi utilizada para dividir a amostra entre: “mais estressados e menos estressados” e “mais resilientes e menos resilientes”.

### Período e Coleta Dos Dados

A partir de grupos religiosos e Organizações Não-governamentais (ONG) que acolhem migrantes internacionais, localizados em três cidades de diferentes regiões de cada um dos países em estudo, é que se iniciou o contato com os potenciais participantes. Tais entidades, em suas sedes, prestam variados serviços a esta população e os pesquisadores inseriram-se nesses contextos para abordar e convidar os migrantes para participar do estudo.

A coleta de dados, conduzida por um grupo de pesquisadores previamente treinados, ocorreu entre os meses de janeiro e setembro de 2022, por meio de entrevistas estruturadas, sempre conduzidas em português ou espanhol e realizadas presencialmente na sede das ONG ou no domicílio dos migrantes. Ao final de cada entrevista o participante era convidado a indicar um ou mais migrantes que conhecia, fornecendo endereço e/ou contato de telefone. Os novos potenciais participantes eram contatados, preferencialmente via telefone, para apresentação dos objetivos do estudo, verificação dos critérios de inclusão e convite para a participação. Caso aceitassem, era agendada uma visita domiciliar em dia e horário oportunos.

Assim, a seleção dos participantes caracterizou-se como não probabilística, por conveniência de acesso dos pesquisadores, do tipo bola de neve. Tendo em vista a limitação de tempo e de recursos humanos e financeiros para prosseguir com a coleta de dados, a amostra chegou a 403 indivíduos.

### Tratamento Dos Dados e Análise Estatística

Todas as informações foram digitadas em uma planilha do Excel para Windows 2010^®^ e após dupla checagem exportadas para o programa estatístico SPSS, versão 22, para processamento das análises. O teste de Alfa de Cronbach demonstrou que a Escala de Resiliência (0.933) foi considerada “excelente” e a Escala do Estresse Percebido (0.712) “aceitável” para a amostra investigada.

A partir dos testes de normalidade Kolmogorov-Smirnov e Shapiro-Wilk, verificou-se que os dados seguiam uma distribuição normal. Para identificar as diferenças entre as médias foram empregados os testes T de Student e teste de análise de variância (ANOVA), sendo realizado o teste de pós-hoc de Sidak para avaliar as comparações múltiplas que foram estatisticamente significantes nos testes de diferença de médias. O intervalo de confiança foi de 95% e o nível de significância adotado foi de 5% para todos os testes (p ≤ 0,05).

### Aspectos Éticos

A investigação desenvolveu-se em conformidade às diretrizes de ética em pesquisa e seu projeto submetido à aprovação por comitês de avaliação nos três países (Brasil: 4.450.114; Espanha: 2756-N21; Portugal: 001/2021). Os pesquisadores asseguraram a livre participação, o sigilo das informações e o anonimato dos entrevistados que assinaram o Termo de Consentimento Livre e Esclarecido em duas vias.

## RESULTADOS

Participaram 403 migrantes internacionais. A maior parte era do sexo feminino (65, 0%); com idades entre 35 e 59 anos (50, 2%); escolaridade superior a oito anos de estudo (78,5%); tinha companheiro (54, 4%); apresentava renda familiar *per capita* mensal de mais de 100 euros (61, 3%); era proveniente da América do Sul (58, 6%); vivia no Brasil (40, 4%); possuía ocupação laboral remunerada (60,8%); se autodeclarou negro/pardo (41, 2%); coabitava com até quatro pessoas (65, 8%) e era de religião católica/cristã (64,8%). Destaca-se que 84, 9% havia imigrado há, no máximo, cinco anos (Tabela 1), com média de 28 meses em Portugal, 30,1 meses no Brasil e 35, 3 meses na Espanha.

**Tabela 1:** Distribuição das variáveis segundo médias de estresse percebido e resiliência entre migrantes internacionais no Brasil, Espanha e Portugal, 2022.

VARIÁVEIS	N	%	Estresse Percebido	p-valor	Resiliência	p-valor
**Sexo**						
Feminino	262	65,0	24.64	0,688	137.87	0,089
Masculino	141	35,0	24.95		133.73	
**Idade**						
18–34 anos	192	47,6	24.77	0,590	135.49	0,352
35–59 anos	202	50,2	24.75		137.54	
60 ou mais anos	09	2,2	24.22		134.77	
**Escolaridade**						
Até 8 anos de estudo	87	21,6	23.88	0,102	142.35	<0,001*
Mais de 8 anos de estudo	316	78,5	24.98		134.81	
**Estado civil**						
Solteiro	160	39,7	24.88	0,733	139.48	0,004*
Casado/União Estável	219	54,4	24.63		137.68	
Divorciado	19	4,7	24.99		125.89	
Viúvo	05	1,2	24.43		138.80	
**Renda familiar per capita**						
Até 100 euros^†^	156	38,7	24.60	0,889	141.14	0,003*
Mais de 100 euros	247	61,3	24.83		133.10	
**Nacionalidade**						
América do Sul	236	58,6	24.54	0,155	137.04	<0,001*
África	80	19,8	24.83		136.71	
Ásia	03	0,7	34.00		99.33	
Europa	35	8,7	25.37		127.22	
América Central	47	11,7	26.35		144.23	
América do Norte	02	0,5	24.28		88.00	
**País de recepção**						
Brasil	163	40,4	25.54	<0,001*	137.22	<0,001*
Espanha	140	34,7	22.72		144.80	
Portugal	100	24,8	26.28		123.46	
**Tempo de migração**						
Até 5 anos	342	84,9	25.96	<0,001*	136.92	0,090
Mais de 5 anos	61	15,1	21.07		133.70	
**Ocupação**						
Desempregado	91	22,6	24.89	0,903	135.58	0,713
Empregado/autônomo	245	60,8	24.79		137.18	
Do lar/estudante	67	16,6	24.38		134.89	
**Cor da pele**						
Negro/Pardo	166	41,2	26.18	0,006*	148.46	<0,001*
Branco	163	40,4	24.12		135.39	
Indígena	61	15,1	22.98		147.65	
Amarelo	13	3,2	22.46		132.40	
**Número de coabitantes**						
Até 4 pessoas	265	65,8	24.72	0,815	135.90	0,125
5 ou mais pessoas	138	34,2	24.80		137.47	
**Religião**						
Catolicismo/cristianismo	261	64,8	24.31	0,001*	139.67	<0,001*
Nenhuma	86	21,3	26.91		122.08	
Ortodoxa	39	9,7	21.91		145.66	
Islamismo	12	3,0	23.25		146.15	
Outras	05	1,1	23.60		137.00	

*P-valor significativo no Teste de T-Student (duas categorias) ou análise de variância (três categorias ou mais);

^†^cotação do euro em 27/12/2023: R$5,37.

Identificou-se que o estresse percebido foi significativamente maior entre migrantes que viviam no Brasil ou em Portugal (p < 0,001); tinham imigrado há menos de cinco anos (p < 0,001); possuíam cor da pele parda/negra (p: 0,006); e referiram não ter religião (p < 0,001).

A resiliência foi maior entre migrantes com até oito anos de estudo (p < 0,001); solteiros, casados ou viúvos (p: 0,004); que possuíam renda familiar *per capita* mensal de até 100 euros (p: 0,003); que viviam na Espanha (p < 0,001); e originários da América do Sul, América Central e África (p < 0,001). Ainda, migrantes de origem europeia tiveram maior média de resiliência comparado com aqueles da América do Norte e da Ásia (p < 0,001).

Os migrantes de raça/cor negra/parda e de origem indígena tiveram maior resiliência comparados com migrantes de cor da pele amarela (origem asiática) e brancos (p < 0,001). Por fim, identificou-se que aqueles que declararam ter alguma religião apresentaram maiores médias de resiliência (p < 0,001).

## DISCUSSÃO

O estudo identificou que diferentes variáveis estiveram relacionadas com maiores níveis de estresse percebido e de resiliência entre migrantes internacionais. O fato de ter sido demonstrado que indivíduos residentes na Espanha eram mais resilientes e os que viviam no Brasil e em Portugal tinham maiores níveis de estresse chama atenção, já que dados do Grupo MIPEX - *Migrant Integration Policy Index* indicam que desses três países, a Espanha é o que apresenta piores índices de integração do migrante, com exceção do acesso aos serviços de saúde^(22)^. Entretanto, os mesmos dados indicam que, no Brasil, aspectos como acesso à educação, saúde e participação política ficam muito abaixo da média global^(22)^, o que sabidamente aumenta o estresse^(9-11)^. Ainda em Portugal, identifica-se um importante discurso de ódio/xenofobia envolvendo migrantes. Dados do projeto MigraMyths apontaram que mais de 75% dos migrantes entrevistados que viviam em Portugal já haviam sofrido preconceitos e estereótipos ligados à migração^(23)^. Além disso, o crescimento da direita radical populista no Brasil e em Portugal (diferentemente da Espanha até o momento), também tem sido apontado como fator relevante na disseminação de discursos de ódio contra minorias raciais e étnicas, que pedem inclusive a expulsão de grupos considerados estrangeiros^(23)^. Diante disso, é de se supor que a ausência de um contexto político que exponha os migrantes a situações estressoras, associada a um maior tempo médio de permanência no país de acolhimento, possam estar contribuindo para uma melhor integração e também para o desenvolvimento da resiliência entre aqueles que vivem na Espanha.

Nesse sentido, a necessidade de adaptação rápida e intensa ao novo ambiente pode justificar o fato de o estresse percebido ter sido maior entre os que tinham imigrado há menos de cinco anos. Uma pessoa que migra para um novo país enfrenta uma variedade de mudanças e obstáculos, incluindo adaptação à nova cultura, barreiras linguísticas, busca por emprego, aquisição de moradia e formação de novas redes sociais^(24)^. As pessoas também podem experimentar sentimentos de isolamento, solidão e incerteza por estarem num ambiente desconhecido e frequentemente longe de suas redes de apoio familiar e social^(25)^. Além disso, a falta de familiaridade com o sistema legal, a burocracia, a dificuldade de se inserir no mercado de trabalho e a restrição de acesso aos serviços básicos podem causar estresse^(26)^.

Os dados do presente estudo demonstraram que pardos e negros se percebiam mais estressados e ao mesmo tempo mais resilientes. Além disso, migrantes originários da América do Sul, América Central e África também apresentaram maiores níveis de resiliência. Esses dados podem sobrepor-se tendo em vista que os migrantes dessas regiões são, em sua maioria, de raça negra^(4,19)^. Maior exposição à xenofobia associada ao preconceito racial podem funcionar como desencadeadores de estresse e, na mesma medida, levarem o indivíduo a lançar mão de estratégias para superar as dificuldades, percebendo-se, então, como mais resilientes aos inúmeros enfrentamentos que passam durante o processo de integração à nova sociedade^(4,5)^.

Sabidamente, a resiliência é influenciada pela cultura^(14)^. Desse modo, a percepção de apoio social de acordo com o local de origem dos migrantes pode ser devida à experiência cultural e ao tipo de rede familiar constituída. No estudo em tela os latino-americanos foram os mais resilientes, possivelmente porque pertencem a culturas coletivistas-horizontais, nas quais a sociabilidade entre iguais é promovida em comparação com outras culturas mais individualistas, que tendem a apresentar valores mais baixos de apoio familiar e social^(27)^.

Destaca-se também que algumas culturas latinas e africanas têm costumes e valores que consideram a família, a comunidade e a solidariedade como estratégias para a superação de desafios^(4,5)^. Os migrantes podem usar esses princípios como uma base social e emocional para suportar as adversidades. Devido a problemas econômicos, políticos, conflitos ou outras circunstâncias, um grande número de migrantes latinos e africanos deixa seus países de origem em busca de melhores perspectivas^(1,2)^. Frente à necessidade de sobreviver e garantir um futuro melhor para si e para as suas famílias, eles tendem a desenvolver a resiliência^(5)^.

No entanto, é fundamental evitar demasiadas generalizações e não considerar a resiliência como uma característica universal para todos os migrantes latinos e africanos. A experiência dos migrantes internacionais é diferente para cada pessoa e a resiliência não é uniforme entre todos, independentemente de sua origem étnica ou cultural. Além disso, uma variedade de características individuais e contextuais pode influenciar a resiliência, já que ela não é genética e sim uma habilidade relacionada ao contexto^(13,14)^.

Identificou-se, no presente estudo, que os divorciados eram menos resilientes. O fato de os casais passarem por um divórcio, o que quebra a estrutura familiar, pode constituir fator para serem menos resilientes, ainda mais se este fato ocorrer no país para onde migraram. Além de todos os desafios da migração e asilo num novo país, ter que viver outra transição familiar, que cria uma nova ruptura com as interações estabelecidas, os papéis anteriores e a rede familiar e social de suporte, pode dificultar o desenvolvimento da resiliência^(28)^.

Já se estabeleceu na literatura a conexão entre melhores resultados de adaptação dos migrantes e a presença de uma adequada funcionalidade familiar. Por exemplo, a participação das famílias migrantes na educação de seus filhos demonstrou ser benéfica para o sucesso acadêmico deles^(29)^. Esta ajuda mútua pode ser especialmente útil para pessoas que enfrentam problemas familiares como conflitos e divórcio, bem como a discriminação ou outros obstáculos à integração. No entanto, é importante observar que nem todas as famílias migrantes têm os mesmos recursos ou oportunidades de contribuir positivamente para seus resultados^(30)^. As experiências das famílias migrantes podem ser moldadas por variáveis como posição social e econômica, habilidades linguísticas e acesso às redes de apoio social. É fundamental que profissionais de saúde e gestores levem em consideração essas diferenças ao criar políticas, programas ou intervenções que busquem promover cuidados às comunidades de migrantes.

Por fim, os resultados do presente estudo sugerem que ausência de religiosidade se relaciona ao maior nível de estresse e ao menor nível de resiliência. Apesar das visões e opiniões divergentes de muitos cientistas e profissionais de saúde mental, as investigações que examinam a religião, a espiritualidade e a saúde expandiram-se rapidamente^(31)^.

Diferentes pesquisas de métodos mistos que investigaram a resiliência em migrantes mexicanos^(14)^, latinos^(16)^ e africanos^(5)^ residentes nos EUA identificaram que os migrantes mais resilientes eram aqueles que tinham altos níveis de religiosidade e utilizavam a fé como estratégia de enfrentamento para dar sentido às suas dificuldades e sofrimentos. Analogamente, investigações quantitativas realizadas com 128 migrantes latinos nos EUA^(15)^ e com 154 jovens migrantes marroquinos na Espanha^(20)^ mostraram que a resiliência esteve diretamente relacionada com a religiosidade. Nesse ínterim, fica evidente que a promoção do apoio social e da religião nas comunidades de migrantes pode melhorar o bem-estar, aumentando a resiliência e reduzindo o estresse e o sofrimento.

Este estudo multicêntrico avança no conhecimento ao demonstrar que migrantes internacionais, em diferentes contextos, vivenciam o estresse percebido, mas, em contrapartida, são capazes de se tornar resilientes. Tais achados podem auxiliar gestores e profissionais de saúde e da assistência social a pensar políticas públicas que busquem a integração do migrante internacional à sociedade, considerando o perfil do migrante que mais percebe o estresse e que, por conseguinte, necessita desenvolver mais fortemente a resiliência. Então, os enfermeiros podem beneficiar-se da elaboração de estratégias de intervenção focalizadas, especialmente, para migrantes que estão há menos tempo no país anfitrião, que possuem a cor da pele preta ou parda e que não têm religião, a fim de promover a resiliência a partir do fortalecimento da saúde mental, do desenvolvimento da capacidade de adaptação a estressores e mudanças e da aprendizagem/utilização do autocontrole, positividade e otimismo.

Por outro lado, reconhece-se que a investigação apresenta limitações. Uma delas refere-se à utilização de uma amostragem não probabilística, a partir de três cidades de cada país participante. Outra se relaciona com o fato de mais de 70% dos entrevistados serem originários da América Latina. Tais características circunscrevem os resultados encontrados e, por isso, sugere-se cautela na comparabilidade com outros cenários e contextos de migração.

De toda forma, os achados são importantes ao permitir que os profissionais de saúde apoiem e melhorem os esforços das organizações na defesa e incentivo à formulação de legislação e políticas visando reduzir o estresse e aumentar a resiliência das pessoas migrantes. Os decisores políticos devem concentrar-se em recursos adicionais para melhorar o bem-estar físico e mental dos migrantes internacionais. Isso tem potencial para impactar sobre a saúde, melhorando sua inserção e integração à sociedade e reduzindo a utilização de serviços de saúde, o que coopera para a sustentabilidade dos sistemas de saúde dos países de acolhimento^(14)^. Com base no diagnóstico levantado, estudos futuros deverão preocupar-se em desenvolver intervenções focadas em reduzir o estresse e promover a resiliência das pessoas e das famílias que vivenciam o processo migratório.

## CONCLUSÃO

Os resultados deste estudo permitiram identificar que as variáveis relacionadas ao maior nível de estresse percebido foram: viver no Brasil ou em Portugal; tempo de permanência no país anfitrião menor que cinco anos; ser negro ou pardo; e não ter religião. Já as variáveis associadas à maior resiliência foram: não ser divorciado; ter até oito anos de estudo; ter renda familiar *per capita* mensal de até 100 euros; ser originário da América Central, América do Sul e África; viver na Espanha; e ter alguma religião.
